# Old friends meet a new foe: A potential role for immune-priming parasites in mitigating COVID-19 morbidity and mortality

**DOI:** 10.1093/emph/eoaa037

**Published:** 2020-10-20

**Authors:** Tara J Cepon-Robins, Theresa E Gildner

**Affiliations:** Department of Anthropology, University of Colorado Colorado Springs, Centennial Hall 120, 1420 Austin Bluffs Parkway, Colorado Springs, CO 80918, USA; Department of Anthropology, Dartmouth College, Silsby Hall, 3 Tuck Drive, Hanover, NH 03755, USA; Department of Anthropology, Washington University, Campus Box 1114, One Brookings Drive, St. Louis, MO 63130, USA

**Keywords:** COVID-19, Hygiene Hypothesis, Old Friends Hypothesis, soil-transmitted helminths, biocultural perspectives, mismatch paradigm

## Abstract

The novel virus, Severe Acute Respiratory Syndrome Coronavirus 2 (SARS-CoV-2), and the associated Coronavirus Disease 2019 (COVID-19) represent a pathogen to which human beings have limited to no evolved immune response. The most severe symptoms are associated with overactive inflammatory immune responses, leading to a cytokine storm, tissue damage, and death, if not balanced and controlled. Hypotheses within Evolutionary Medicine, including the Hygiene/Old Friends Hypothesis, provide an important lens through which to understand and possibly control this overactive immune response. In this article, we explore the role that infection with soil-transmitted helminths (STHs; i.e. intestinal parasitic worms) may play in dampening SARS-CoV-2 symptoms and mitigating the worst COVID-19 outcomes. Specifically, STHs stimulate the immunosuppressive and regulatory T-helper 2 (T_H_2) branch of the immune system, which decreases ACE2-receptor expression (i.e. receptors SARS-CoV-2 uses to infect host cells), balances the inflammatory T_H_1/T_H_17 branches of the immune system triggered by SARS-CoV-2 infection, and reduces inflammation through the release of anti-inflammatory/regulatory cytokines. Because STHs are common and affect the most vulnerable and marginalized members of society, it is especially important to consider how these parasites may impact COVID-19 outcomes. Areas experiencing endemic STH infections are often characterized by a lack of preventative infrastructure and medical care, which may further exacerbate risk of SARS-CoV-2 infection and COVID-19 development. For this reason, we also explore biocultural factors that contribute to disease outcomes for both SARS-CoV-2 and STH infections. Biocultural and Evolutionary Medicine perspectives on COVID-19 are crucial for understanding the global impact of the disease.

Lay summary: An evolutionary perspective is required to understand the global impact and various presentations of COVID-19. We consider how coinfection with soil-transmitted helminths (common parasitic worms that coevolved with humans) may suppress inflammatory immune activity, thereby potentially reducing COVID-19 disease severity. Structural and lifestyle factors shaping coinfection patterns are also discussed.

## INTRODUCTION

The novel virus, Severe Acute Respiratory Syndrome Coronavirus 2 (SARS-CoV-2), and the associated Coronavirus Disease 2019 (COVID-19), spread rapidly among the global population. By September 6, 2020, an estimated 26 962 795 people have tested positive for infection and 880 955 have died [1]. However, current evidence suggests that the risk of morbidity and mortality from COVID-19 varies across individuals and populations, with certain demographic groups (e.g. older adults and those with pre-existing chronic conditions) and countries (e.g. the United States, Brazil, India, Russia) exhibiting higher infection rates and/or worse disease outcomes [1]. Most COVID-19 studies to date have focused on proximate disease determinants and risk factors [2–4]; however, we contend that it is also important to examine SARS-CoV-2/COVID-19 through an evolutionary lens.

In this theory-based review, we consider both proximate mechanisms and ultimate causes of COVID-19 virulence, arguing that an Evolutionary Medicine framework should be used to examine how mismatches between our evolved biology and current lifestyles may shape COVID-19 disease outcomes. Because COVID-19 is associated with a hyperinflammatory immune response, we focus this paper on the immunoregulatory effects of certain parasitic species, so-called ‘old friends’, that share long coevolutionary histories with humans [5–7] and are known to dampen harmful inflammatory responses [8], such as those observed in COVID-19.

Specifically, we highlight the need for an evolutionary perspective to clarify how variation in parasitic disease exposure throughout the life course and across populations may affect immune system regulation, influencing immune-reactivity to SARS-CoV-2 and possibly altering COVID-19 severity. We use the mismatch paradigm to consider how evolutionarily novel environments that alter parasite exposure and subsequent immune development may result in more robust inflammatory immune responses, possibly increasing the risk of severe COVID-19 symptoms. This approach allows for the application of a biocultural perspective to identify shared socio-ecological factors influencing both parasite and SARS-CoV-2 infection patterns, as well as COVID-19 disease outcomes. We end with a discussion of future work needed to elucidate the relationship between parasite infection and COVID-19 disease outcomes.

An exploration of potential interactions between macroparasite infection and immune responses to SARS-CoV-2 is timely and of great importance. Coinfection between parasites and COVID-19 is occurring in many locations around the world, especially in regions with limited access to medical intervention, and coinfection prevalence will likely increase as basic medical services and government resources needed for anti-helminth public health campaigns are depleted by the ongoing fight against COVID-19. This information may help healthcare workers and researchers better understand and track various possible outcomes of SARS-CoV-2 infection.

## KNOWN COVID-19 RISK FACTORS

The disease COVID-19 affects multiple organ systems, resulting in major respiratory distress, as well as potentially impacting the blood vessels, gastrointestinal tract and central nervous system [9–11]. Current evidence suggests that up to 81% of infected individuals may experience few to no symptoms [12–14], while mortality rates in the 20 most affected countries are highly variable depending on country (e.g. 2% in the Philippines and South Africa; 3% in Brazil and the United States; 6% in Spain; 11% in the Mexico) [15]. The range and variation in disease outcomes suggests that there are individual- and population-level factors at play that shape COVID-19 prognosis, including pre-existing health conditions.

Recent evidence has clearly linked obesity, cardiovascular disease, autoimmune diseases, and other chronic diseases with more severe COVID-19 symptoms and an increased risk of hospitalization and death from infection [2, 4, 16]. Importantly, most comorbidities linked with COVID-19 symptoms are associated with pro-inflammatory immune activity, suggesting that an overreactive inflammatory response may be a primary driver of disease severity [17, 18]. Moreover, regardless of pre-existing conditions, COVID-19 patients with the worst health outcomes typically exhibit an overreactive inflammatory response, called a cytokine storm—a severe immune reaction initiated by the release of too many cytokines over a short period of time, leading to extensive host tissue damage—and hypercoagulation [17, 18].

Age also appears to be an important player in COVID-19 disease outcomes, such that mortality rates are highest among older adults [2]. Children may be just as likely to become infected, but are much less likely to develop severe symptoms [19], which could be attributable to immune priming from repeated viral exposures common among young children [3], anti-inflammatory immune profiles common during early life [20] or the immaturity of cellular receptors to which SARS-CoV-2 binds (i.e. angiotensin-converting enzyme [ACE] 2) [21]. While uncommon, recent evidence suggests children are not completely protected from the harmful effects of COVID-19-related hyperinflammatory responses, with some previously infected children later developing potentially lethal Multisystem Inflammatory Syndrome [22]. Still, age profiles of severe COVID-19 point to the importance of individual immune system variation, especially regarding regulatory and inflammatory processes.

Population-level differences in disease patterns are also apparent. While political and cultural factors certainly shape the effectiveness of pandemic control measures, underlying differences at the local level (e.g. population density, hygiene/sanitation, education about disease avoidance, prevalence of other diseases) and subsequent variation in immune system development early in life may also play important roles in shaping chronic inflammatory risk and immune response to SARS-CoV-2. An evolutionary perspective is consequently required to clarify why certain individuals and populations may be at a greater risk of experiencing severe COVID-19 outcomes.

## AN EVOLUTIONARY HYPOTHESIS FOR UNDERSTANDING COVID-19 OUTCOMES

### Evolutionary mismatch: the Hygiene and old Friends Hypothesis

The field of Evolutionary Medicine offers an important lens through which to study COVID-19 patterns. Evolutionary (or Darwinian) Medicine addresses how natural selection has shaped the human body, leading to beneficial traits, trade-offs and seemingly suboptimal physical designs [23–25]. In other words, Evolutionary Medicine moves beyond asking proximate questions about how the human body works and what causes sickness to examine ultimate explanations regarding how natural selection and other evolutionary forces have resulted in vulnerabilities that predispose individuals to injury and illness [24–27]. One paradigm within Evolutionary Medicine explores how ‘environmental mismatches’ between ancestral environments (which shaped human evolution) and current environmental conditions increase disease risk. One example within the mismatch paradigm, the Hygiene Hypothesis, contends that high levels of sanitation in many high-income regions lead to reduced pathogen exposure during key developmental periods; this lack of immune stimulation by evolutionarily relevant pathogens is thought to result in immune dysregulation and associated increases in chronic inflammation, allergy and autoimmunity [28–33].

A counterpart to the Hygiene Hypothesis, the Old Friends Hypothesis, more narrowly focuses on the coevolutionary relationship humans share with certain commensal bacteria and macroparasite species, pointing to a specific branch of our immune system (i.e. Type 2 [T_H_2] immunity), which evolved specifically in response to macroparasite infection [5–7]. According to this hypothesis, relatively recent medical, hygiene and sanitation advances limit exposure to these ‘old friends’, resulting in immune dysregulation that favors pro-inflammatory pathways and causes the body to overreact to harmless or self-produced stimuli. This dysregulation ultimately contributes to the high rates of chronic inflammatory diseases (e.g. allergies, autoimmune diseases, cardiovascular disease) seen in high-income regions [5, 29, 34].

### Helminths and coevolutionary mechanisms favoring immunosuppression

There are many types of macroparasites that infect humans. Here we highlight a group of parasitic intestinal worms called soil-transmitted helminths (STHs) due to their global prevalence and coevolved immunoregulatory effects. STHs are the most prevalent neglected tropical disease (i.e. a group of bacterial, viral and parasitic diseases found in areas of extreme poverty) worldwide [35]. Notably, STHs infect more than a quarter of the global population, with millions of individuals infected with a few key species: ascarids (∼800 million cases globally), whipworm (∼400 million cases globally) and hookworm (∼400 million cases globally) [36]. While often asymptomatic or mild, severe STH infection can result in nutritional deficiencies, mental and developmental delays, organ failure and death [37]. Because STHs are widespread and predominantly impact vulnerable low-income populations with limited healthcare access, they represent an important group of infections to consider in conjunction with COVID-19 outcomes.

Like other parasites, STHs survive in their host through balanced parasitism in which they actively manipulate host immune activity to tolerate the parasite’s presence [37, 38]. This balanced parasitism reduces the risk of harmful symptoms for the host, because an overreactive immune response to such large pathogens would lead to pervasive host immunopathology [8]. For instance, when hosts fail to downregulate inflammation in response to *Tania solium* (a tapeworm helminth, but not an STH) cysts in the brain (i.e. cysticercosis), infection results in seizures and death; yet, while dampened immune activity decreases immunopathology, increased host tolerance allows the cysts to proliferate [8, 39, 40]. Thus, a balanced immune response that allows both the parasite and host to survive represents an incredibly complex and nuanced example of coevolution. Researchers continue to examine the complicated ways by which STHs hijack host immune systems. During primary infection, STH antigens and derived products secreted directly from the parasite trigger the T_H_2 response [37, 41, 42]. When the T_H_2 response is triggered by parasites, immune cell profiles become more anti-inflammatory. Intracellular microbial and viral infections, on the other hand, trigger inflammatory T_H_1 and T_H_17 responses.

More specifically, helminth antigens are able to actively suppress inflammation by suppressing the production of interleukin (IL)-12 and by blocking the induction of the T_H_1 pathway [8]. This helminth immunoregulation ultimately leads to asymptomatic infections favoring the production of immunoregulatory or anti-inflammatory cytokines, including IL-4, IL-10, IL-13, IL-33 and transforming growth factor beta (TGF-β) [8, 43]. Altogether, STH infection generally increases production of CD4+ T cells, Treg cells and Breg cells and results in the death of T_H_1 immune cell types [43, 44]. Interleukin-10, IL-4 and IL-13 are especially important to host tolerance of helminth infection because IL-10 is anti-inflammatory, while IL-4 and IL-13 are involved in the repair of tissue damage caused by STH migration through the skin (hookworm) and lungs (hookworm and ascarids), as well as damage to intestines when STHs feed directly (hookworm and whipworm) (Table 1) [8].

**Table 1.** Species-specific host relationships and demonstrated cytokine associations for three prevalent STHs^a^

Infection type	Locations/relationships in the body	Cytokine associations	References
Ascarids (e.g. *Ascaris lumbricoides*)	Migrate through the liver and lungs Mature and passively feed in the small intestine	Increased: IL-4^b^ IL-5 IL-10 Decreased: IL-6 IL-12 TNF-α IFN-γ	[37, 45–48]
Whipworm (e.g. *Trichuris trichiura*)	Mature and directly attach/feed in the large intestine	Increased: IL-4 IL-9 IL-10 IL-13 TNF-α *IFN-γ* Decreased: IFN-γ	[37, 47,49, 50]
Hookworm (e.g. *Ancylostoma duodenale, Necator americanus*)	Enter through the skin Migrate through the lungs Mature and directly attach/feed in the upper small intestine	Increased: IL-4 IL-5 IL-9 IL-10 IL-13 TGF-β Decreased: IL-6 IL-17 TNF- α IFN-γ	[37, 51–53]

a This table represents a generalized overview, but it is important to note that these relationships are complex. For instance, the immune response to STHs appears to vary by age, as well as duration and intensity of STH infection. Because different STHs affect different parts of the body, cytokine levels and effects may also vary by sampling location and sampling techniques.

b Underlined cytokines are associated with immunosuppressive and/or anti-inflammatory activity, while italicized cytokines are associated with increased immune activity and/or pro-inflammatory effects. While this represents the most commonly discussed effects of each cytokine, it is important to note that some cytokines can vary in their activity (e.g. anti- vs pro-inflammatory) depending on context.

Soil-transmitted helminths are important to consider in conjunction with COVID-19 because they affect many of the same organ systems. For instance, many STH species affect the lungs, with some migrating through lung tissue during their larval stage [37, 38]. Further, STHs also impact the intestines and the overall immune system [37]. Cumulatively, chronic STH infections promote immunotolerance, immunoregulation and tissue healing while suppressing inflammation.

### A world without STHs: immune dysregulation and hypersensitivity

**Figure 1.**
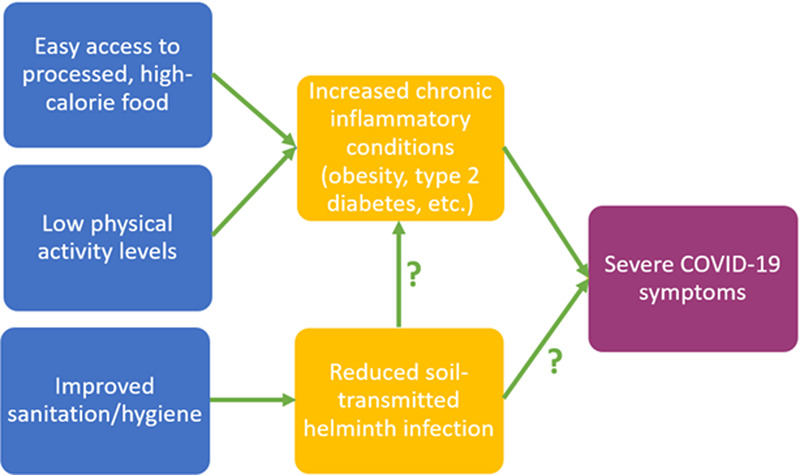
Lifestyle factors linked with elevated rates of chronic inflammatory conditions and reduced rates of STH infection. The physiological effects of decreased STH infection are hypothesized to contribute to both increased chronic inflammation and the risk of severe COVID-19 symptoms, while chronic inflammatory conditions have been identified as risk factors for worse COVID-19 disease outcomes

Many common chronic inflammatory conditions (e.g. autoimmune diseases, cardiovascular disease, obesity, age-related chronic inflammation) are considered mismatch diseases prevalent in higher-income countries. This pattern is likely due to multiple lifestyle factors (Fig. 1), including lower physical activity levels, unhealthy diets and reduced exposure to important immune priming pathogens like STHs [8, 54]. The processes that STHs use to trigger immunotolerance and reduce inflammation are very similar to the homeostatic processes that promote self-tolerance, as well as tolerance for harmless environmental antigens (allergens) and commensal bacteria [8]. The loss of immune priming from STH infection is therefore thought to result in allergic and autoimmune responses. For instance, without helminths to promote the T_H_2/Treg profile, tolerance for commensal bacteria and certain food types may be lost and conditions like inflammatory bowel disease and Crohn’s disease may occur [8]. *Trichuris trichiura* (whipworm) and its close relative *Trichuris suis* (pig whipworm) have been shown to reduce intestinal inflammation due to excreted products that trigger a localized T_H_2/Treg response [42, 53, 55, 56].

Importantly, evidence suggests that STH-related immune activity is linked with allergy risk. An overactive T_H_2 immune pathway is associated with increased allergic sensitivity, suggesting that the immune pathway that initially evolved in response to macroparasites becomes dysregulated and pathogenic when triggered by non-parasitic antigens [38]. In the case of allergy, it appears that the T_H_2 response is activated without the immunosuppressive and anti-inflammatory cells that helminths also trigger. For example, when helminths bind to dendritic cells and trigger the T_H_2 response, it is balanced by the production of IL-10 and Treg, which ultimately reduce lung inflammation [38]. This balance with regulatory cells is not evident in allergic responses, leading to an overreactive response to harmless antigens.

Beyond localized anti-inflammatory effects, immune priming from STH infection during immune system development in infancy and early childhood may provide systemic protection from inflammation, likely by skewing future immune responses toward adaptive T_H_2-dominated immunity, balancing both T_H_1 immunity and the innate inflammatory response [57]. Exposure to STH infection at key developmental periods may therefore reduce inflammation across the lifespan; however, additional longitudinal work across populations experiencing varied pathogen loads is required to clarify the ages at which STH infection may play the greatest role in shaping later immune function. Additionally, a systemic reduction in inflammation may also occur if an individual is first exposed to STH infection during adulthood, but the extent and benefits of exposure at this later age are unclear [29]. Still, though tentative and preliminary, reduced systemic inflammation related to helminth infection has been linked to prevention and symptom reduction in several inflammatory autoimmune diseases, like multiple sclerosis and rheumatoid arthritis [58, 59].

The potential for STH-associated immune responses to shape the risk of developing chronic inflammatory conditions may prove important in countering COVID-19. While STHs are ‘old friends’, coevolving with humans throughout our long evolutionary histories, novel and more virulent pathogens have emerged in recent human history. Humans have no specific evolved immune responses to these new health challenges, allowing them to readily infect individuals and spread rapidly among crowds [60, 61], as is evident with SARS-CoV-2. Immune responses to the virus may consequently be ineffective or cause collateral damage to host tissue due to excessive inflammation, which may be balanced by coinfection with STHs.

### Soil-transmitted helminths and COVID-19 prognosis

Unlike macroparasite infections, viruses trigger the T_H_1 immune response, which is dominated by proinflammatory cytokines (e.g. IL-6, CRP, TNFα, IL-1β) [62]. In healthy immune systems, both T_H_1 and T_H_2 responses are important and act to balance one another, hypothetically preventing immune dysregulation [63]. These relationships demonstrate a need for Evolutionary Medicine perspectives in understanding COVID-19 disease outcomes. Elevated levels of proinflammatory cytokines IL-6, IL-8 and TNF-α appear to predict patient survival [64] suggesting that imbalanced immune activity resulting from evolutionary mismatches seems to be the primary driver of COVID-19 morbidity and mortality. Clarifying how complex interactions between distinct immune pathways may drive COVID-19 disease progression is critical in the design of effective interventions. In fact, stimuli that trigger the T_H_2 response are being targeted in COVID-19 vaccine development because of the T_H_2 response’s ability to counter the hyperinflammatory T_H_17 response [65].

**Figure 2.**
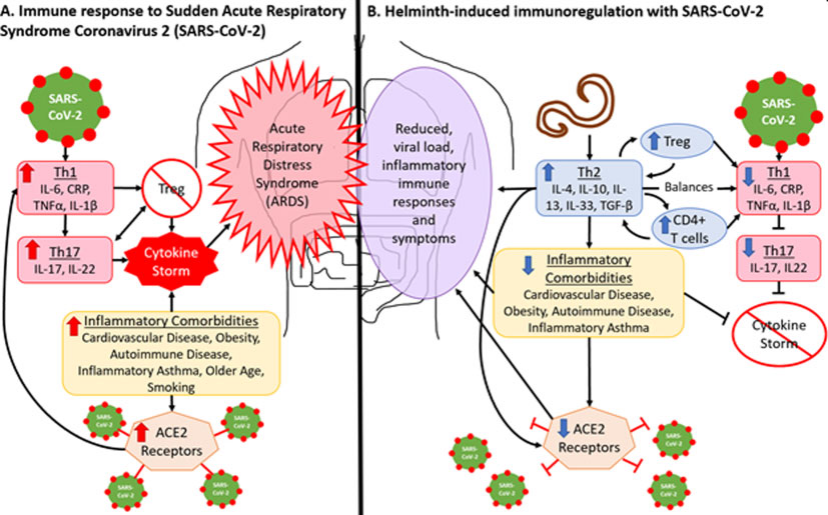
(**A**) SARS-CoV-2 infects the host through angiotensin-converting enzyme (ACE) 2 receptors which are elevated in inflammatory conditions. Infection triggers the T_H_1 response, which produces proinflammatory cytokines. When overproduced, T_H_1 cytokines trigger the hyperinflammatory T_H_17 response [17, 18]. These responses inhibit Treg cell differentiation, which is needed to suppress inflammatory cytokines. A cytokine storm occurs when proinflammatory cytokines proliferate uncontrollably. (**B**) The T_H_2 response to STHs suppresses inflammation through the production of anti-inflammatory and regulatory cytokines, as well as by balancing the T_H_1 pathway through the production of CD4+ T cells and Treg cells [8, 43, 44]. The T_H_17 response is less likely to be induced and the cytokine storm should not occur [66]. STHs are associated with reduction in inflammatory comorbidities [8, 34], further reducing likelihood of a cytokine storm and ACE2 receptor presence [69–71]. The T_H_2 response may also directly reduce ACE2 receptor presence [10, 72]

Figure 2A describes the immune response and disease outcomes associated with acute respiratory distress syndrome caused by COVID-19. This severe inflammatory response results in the development of a ‘cytokine storm’ driven by the T_H_1 and T_H_17 pathways [16], which results in the uncontrolled proliferation of proinflammatory and destructive cytokines [16, 17]. Because of their ability to trigger an immunosuppressive T_H_2 response, which balances and regulates the proinflammatory T_H_1 and T_H_17 responses through the release of regulatory and immunosuppressive cytokines (i.e. IL-4, IL-10, IL-13, IL-33, TGFβ) and Treg cells [6, 8, 43, 44, 63], individuals coinfected with STHs may experience milder COVID-19 symptoms and no cytokine storm [66] (Fig. 2B).

Moreover, evidence indicates that the elevated production of pro-inflammatory cytokines which initiate a cytokine storm is triggered by the activation of pattern recognition receptors [67]. These same receptors are targeted by helminths as part of an immunoregulation strategy that promotes chronic STH infection [43, 68]. Thus, in addition to increasing the production of anti-inflammatory immune cells, STH infection may also act on upstream regulators of pro-inflammatory immune responses, providing another pathway through which helminths may decrease the risk of COVID-19 symptoms, including cytokine storms.

Elevated inflammation in general seems to be associated with the increased presence of host ACE2-receptors, which SARS-CoV-2 uses to infect cells [21]. High levels of these receptors are evident in inflammatory conditions, including cardiovascular disease, diabetes, smoking and inflammatory asthma [69–71]. The T_H_2 response appears to decrease the presence of ACE2-receptors [72]. To take one example, allergic asthma (as opposed to non-allergic asthma), which is triggered by the T_H_2 pathway, has been linked to a reduction in ACE2 expression [72]. These findings have led some researchers to suggest that allergic asthma may offer some protection from severe COVID-19 [72], although this connection is still debated [69]. To our knowledge, no research has directly tested the relationship between STHs and ACE2 expression. Some STHs do stimulate the T_H_2 response in the lungs to promote host parasite tolerance [38], suggesting that STHs have the same effects in the lungs as allergic processes, while simultaneously triggering anti-inflammatory and regulatory immune responses. For this reason, it seems likely that STH presence would also reduce ACE2-receptors on respiratory cells, thereby inhibiting the ability of the SARS-CoV-2 virus to infect the host and reducing viral load, although this idea needs further testing.

Additional aspects of STH infection may also decrease viral load. Infection with STHs results in increased IL-5 production (Table 1), which is associated with eosinophilia (i.e. elevated levels of a type of white blood cells called eosinophils) [73, 74]. Eosinophils target STH larva in the lungs and elicit immunoregulatory and antiviral effects [73, 75]. Early studies on COVID-19 found that eosinopenia (i.e. low to no eosinophils in a complete blood count) was common in those hospitalized with COVID-19 and was associated with higher death rates [76]. Because of their anti-viral effects, eosinophilia caused by STH infection prior to SARS-CoV-2 exposure could work to fight the virus in the early stages of infection and reduce viral load [75]. Reductions in SARS-CoV-2 viral load may strongly shape disease outcomes. Current evidence indicates that high viral loads are positively related to COVID-19 disease severity; specifically, viral loads measured from respiratory samples were significantly higher among individuals with severe COVID-19 symptoms compared to those with mild cases [77–79]. High viral loads were also correlated with elevations in pro-inflammatory cytokines (e.g. IL-6) [77–79]. It is therefore possible that STH-linked reductions in viral loads and pro-inflammatory immune activity may prevent the most severe disease outcomes.

In addition to reducing viral load and mitigating COVID-19-related hyperinflammation, STH infection may also decrease the prevalence of preexisting chronic inflammatory conditions known to be risk factors for COVID-19 morbidity and mortality [5–7, 28–34]. It may be that these risk factors are linked with worse COVID-19 outcomes because they reflect immune systems predisposed to hyperinflammatory responses (i.e. T_H_1, T_H_17) due to the lack of T_H_2 priming during development and altered base-line inflammatory activity. Without STH exposure and T_H_2 priming to balance these inflammatory responses, the overactive T_H_1 and T_H_17 responses may plunge those infected with SARS-CoV-2 into cytokine storms. If true, this suggests that populations with endemic STH infections (and fewer pre-existing chronic conditions) should exhibit better COVID-19 disease outcomes, including fewer cases of fatal cytokine storms.

While very few studies have tested possible links between COVID-19 disease outcomes and co-existing parasitic infection, limited early evidence suggests there may be a meaningful association. For example, one preliminary study, which has not yet been peer-reviewed, compared WHO data on COVID-19 prevalence around the world to data on prevalence of various parasitic infections. The researchers documented inverse correlations between incidence of symptomatic COVID-19 and STH prevalence [80]. They also show that countries with endemic STH infections exhibited significantly lower odds of reporting high COVID-19 levels nationally [80]. These findings provide tentative support for our hypothesis that STHs may improve COVID-19 health outcomes, but this early work does not consider the possible pathways by which parasitic infection may instead worsen COVID-19 symptoms or consider other biocultural factors that may be driving this relationship.

For instance, it is important to recognize that parasites, including STHs, are a major health problem globally. While we hypothesize that the immunosuppressive effects of STH infection may mitigate COVID-19 outcomes, it is possible that the STH-associated immunoregulation may increase SARS-CoV-2 susceptibility (despite the hypothesized T_H_2-associated reduction in ACE2-receptors) and lung damage associated with COVID-19. Helminth-associated immunosuppression has been shown to reduce the likelihood of fighting off infection quickly, as is the case with coinfections between helminths and the bacteria *Mycobacterium tuberculosis* [8, 81]. Evidence also indicates that parasite-related immunosuppression reduces vaccine efficacy [82], leading some to suggest that STH infection may inhibit the development of long-term SARS-CoV-2 immunity [83]. Thus, although many aspects of STH infection likely reduce SARS-CoV-2 viral load (as discussed above), it is also possible that STH-linked immunosuppression may inhibit preliminary immune activity, increasing viral load and extending the duration of infection in some instances. Additional work is needed to examine these complex associations.

Still, suppressed immune activity may also prevent the deadliest aspects of COVID-19, despite the possibility of increased infection risk. Reducing COVID-19-related immune activity even moderately may substantially improve individual outcomes. For instance, one recent study found that individuals with asymptomatic SARS-CoV-2 infections mounted weaker immune responses and had cytokine levels comparable to healthy controls [84]. Thus, the difference between asymptomatic and deadly outcomes may lie entirely in how the immune system responds. It is consequently possible that while STH-linked immunoregulation may increase risk of initial SARS-CoV-2 infection, it may also lead to milder symptoms and reduced mortality, as is evident in cases of coinfection between STHs and other deadly pathogens. Coinfections between malaria and STHs, for example, may provide a parallel for understanding how STHs could affect severe COVID-19 risk. Specifically, while helminth infections increase susceptibility to malaria, they reduce risk of hyperinflammatory immune responses and cytokine storms associated with severe malaria [8, 85, 86]. It is possible that STH infections may have a similar effect on SARS-CoV-2 infection and COVID-19 disease outcomes, although this remains to be directly tested.

Another concern is that the immune response to STH infection could make certain COVID-19 outcomes worse. For instance, elevated eosinophil levels caused by STH infection could have proinflammatory affects in host tissue, which could lead to increased lung tissue damage, although pulmonary eosinophilia has not been linked with lung damage in COVID-19 patients to date [75]. This also seems unlikely given that, as mentioned previously, low eosinophil levels appear to be associated with worse COVID-19 outcomes [76]. Additionally, studies have documented high levels of some T_H_2 immune cells in COVID-19 patients (e.g. IL-4 and IL-10) [9, 87]; although it has been suggested that these represent normal immune responses to SARS-CoV-2 tissue damage geared toward regulating inflammation and repairing tissue [88]. Importantly, populations with endemic parasite infections likely exhibit a more robust T_H_2 response and higher eosinophil levels at the start of infection, resulting in comparatively less tissue damage and lower viral loads. However, to the best of our knowledge, no study has directly tested relationships between STH infection and COVID-19 outcomes. Clearly, more work is needed to understand how parasite infection affects COVID-19 disease outcomes, especially across biologically and culturally diverse populations [87, 88].

## BIOCULTURAL PERSPECTIVES FOR UNDERSTANDING COINFECTION AND HEALTH OUTCOMES

**Figure 3.**
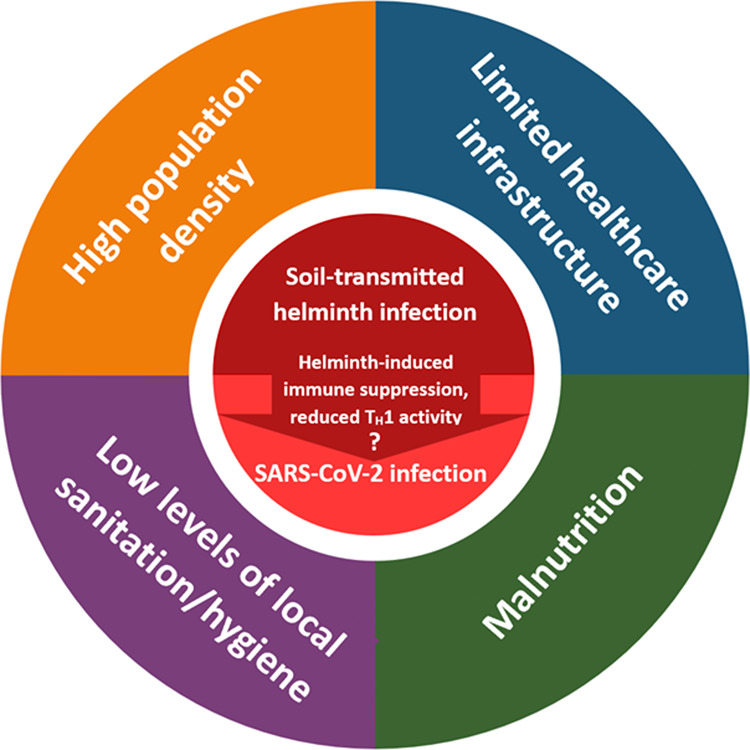
Structural, population-level factors associated with an increased risk for both STH and SARS-CoV-2 infection. These structural factors interact and reinforce one another, contributing to rapid disease spread, increased infection susceptibility, few treatment options, and general poor health. It is expected that STH and SARS-CoV-2 infection risk are both negatively affected by these socio-ecological factors. These two infection types are also hypothesized to interact, such that the immunosuppressive effects of STH infection may increase the risk of initial SARS-CoV-2 infection and make it more difficult to fight off infection

While we hypothesize that coinfection with STHs and SARS-CoV-2 may reduce the risk of severe inflammatory COVID-19, it is important to consider other factors associated with both infection types that may also affect disease outcomes. For instance, STHs tend to be most common in low-income countries that already experience high infectious disease burdens due to limited access to medical care or infrastructure that protects from infection. Thus, variation in disease exposure and subsequent immune activity is likely due to a mix of factors, including individual immune system development (as discussed above), but also local conditions (Fig 3; e.g. population density, healthcare access, sanitation levels, etc.) [83, 89–93]. These interwoven effects may be difficult to disentangle and should be considered together. For example, while high population density may facilitate the spread of infections, additional factors may mitigate this risk (e.g. high levels of local sanitation, cultural norms promoting preventative behaviors, well-funded medical infrastructure with rapid disease screening and treatment). It is therefore necessary to consider how biological, cultural and socioeconomic factors interact to shape STH and SARS-CoV-2 exposure and subsequent health consequences.

### Local infrastructure

Several biocultural factors shape infection risk and disease prognosis for a range of medical conditions. For instance, lack of sanitation infrastructure, poor individual hygiene and limited healthcare access have been associated with numerous parasitic infections [94–96]. To take one example, research among Indigenous Shuar of Amazonian Ecuador indicates that aspects of household construction (e.g. water source and floor material) tied with lifestyle and local sanitation levels are significantly associated with infection risk and intensity for common STH species [94, 96]. Similarly, highly infectious diseases (i.e. bacteria and viruses that spread rapidly through large populations), proliferate under these same conditions, as well as through crowding and high rates of person-to-person contact [60, 61]. This is true of SARS-CoV-2, with early data showing that COVID-19 risk and disease outcomes may be dose dependent and with frontline healthcare workers being at a greater risk due to elevated viral load from regular exposure [97]. This can be extended to suggest that prolonged exposure to infected individuals within confined spaces with lower hygiene/sanitation infrastructure may increase viral load and lead to worse health outcomes.

Additionally, lack of local healthcare infrastructure is expected to exacerbate both STH infection risk and the spread of SARS-CoV-2. For instance, a lack of laboratory resources for infection diagnosis, insufficient diagnostic supplies and protective equipment, and limited access to healthcare providers may all inhibit the prevention, identification and containment of SARS-CoV-2 and STH infections [89–93], especially as local medical resources are exhausted by increasing demands of COVID-19. Beyond impacting measures that prevent disease spread, insufficient healthcare infrastructure also compromises the ability to treat infected individuals. Inadequate access to needed medical equipment, hospital space, trained care providers and medications have been linked with worse health outcomes for both STH and SARS-CoV-2 infections [89–93].

### Nutritional status

Beyond variation in local infrastructure and healthcare access, differences in nutritional status may also impact STH and SARS-CoV-2 infection risk. Chronic malnutrition has been linked with tradeoffs between immune function and other important physiological functions due to the high energetic costs of immune responses to pathogens [83, 98]. For instance, research shows tradeoffs in growth associated with elevated immune activity, demonstrating the high energetic toll of immune responses in nutritionally stressed populations [57, 99]. Research suggests that immune responses to viruses and bacteria, especially those that cause acute inflammation, are extremely energetically expensive. A positive energy balance (i.e. a healthy level of body fat) is therefore very important in buffering against these energetic demands, especially among children [99]. Although it is important to note that high, unhealthy levels of body fat may instead act as a major risk factor for certain infection types, including SARS-CoV-2 [16]. In addition to leading to life history tradeoffs (e.g. between immune function and growth), undernutrition itself may also be immunosuppressive due to a lack of resources needed to sustain energetically expensive immune responses [100, 101].

Helminth infections have also been shown to directly compromise host nutritional status by extracting host nutrients [37, 95]. Further, STH infections are related to increased rates of anemia due to the destruction/consumption of hemoglobin in red blood cells, which is a necessary component of innate immunity [36, 102, 103]. A parasite-induced reduction in host nutritional status further compromises the ability of the host to mount an effective immune response to clear infections with other pathogens. Likewise, poor nutrition may be a risk factor for severe COVID-19 symptoms [83], consistent with the fact that malnutrition appears to have contributed to mortality risk in both the 1918 and H1N1 influenza pandemics [83, 104, 105].

### The intestinal microbiome

Alterations in infrastructure and diet have been associated with variation in the human intestinal microbiota, such that increased sanitation and consumption of processed foods (among other factors) appear to result in reduced diversity [106, 107]. A healthy, diverse microbiota is important for proper immune system development and function [108]. Interestingly, STHs also modify the intestinal microbiota by increasing the abundance of immune-regulating bacterial species that favor chronic STH infection, like those in the family *Lactobacillaceae*, [109]. Further, interactions between STHs and the microbiota have been shown to reduce lung damage from other viral infections by promoting production of microbiota-dependent anti-inflammatory cytokines [110]. Because the microbiota composition and its effects on health outcomes vary based on numerous biocultural factors that extend beyond the scope of this article, future research should consider its role in COVID-19 outcomes in conjunction with STH infections.

Several intersecting biocultural factors influence infectious disease risk (including both STH infections and SARS-CoV-2). These complex interactions are currently understudied in low-income countries and regions, where populations are most vulnerable to rampant infectious and parasitic disease spread. Further, COVID-19 disease prognosis and presentation may differ from the symptoms typically exhibited in patients from high-income nations because of the immunoregulatory effects of STH coinfections. Healthcare workers and researchers should consequently consider how local conditions may influence SARS-CoV-2 spread and COVID-19 symptomology across diverse populations. Clarifying various possible manifestations of COVID-19 is crucial for the correct identification and containment of cases, especially among individuals with comorbidities (e.g. parasitic infection) not typically seen in high-income countries.

## UNANSWERED QUESTIONS

Given the known effects of STH infection on host immune function, it seems probable that pre-existing STH infection may influence SARS-CoV-2 infection risk, as well as COVID-19 disease severity. Still, given the negative health effects associated with heavy STH infection (e.g. nutritional deficiencies, stunted growth, impaired physical and cognitive function, organ failure) [36, 37], we are not advocating for the use of STHs as a viable strategy to prevent severe COVID-19 symptoms (e.g. through intentionally exposing naïve hosts to STH infection). Moreover, the hypothesized relationships between STH infection and COVID-19 outcomes remain poorly tested. Further testing of these relationships is necessary to clarify all possible manifestations SARS-CoV-2 infection across populations facing varied pathogen loads. Here we outline some of the complex issues that should be addressed in future studies:

### Are there measurable benefits to the immunoregulatory effects of STH infections in individuals coinfected with SARS-CoV-2?

Work needs to be done to understand if COVID-19 symptom severity is significantly reduced among individuals coinfected with STHs compared to those without a concomitant STH infection (i.e. by comparing rates of diagnosed symptomatic COVID-19 cases between areas with and without endemic STH infection). In addition, within a population experiencing endemic STH infection, links between current STH infection and the likelihood of severe COVID-19 symptomology should be considered in association with deworming programs, and studies could be designed to test and monitor outcomes between individuals enrolled in deworming programs and those who have not recently received anthelminthic treatment. If STH infection appears to mitigate COVID-19 severity, the immune mechanisms accounting for this difference should be explored, including a hypothesized reduction in ACE2 receptors and altered cytokine profiles in STH-infected versus uninfected COVID-19 patients. Research also needs to address if the immunoregulatory effects of STH infections vary across individuals with other pre-existing comorbidities (e.g. obesity, poor nutrition, anemia, etc.) that may be related to their immune state.

### Do specific species or types of STHs have differential effects on SARS-CoV-2 susceptibility and COVID-19 outcomes?

The STH categorization contains multiple species that occupy different parts of the digestive tract, interact with their host in different ways and may be associated with species-specific cytokine profiles (Table 1). This means that STH effects on COVID-19 disease outcomes may vary by species, as well as timing, duration and intensity of infection. For instance, both ascarid and hookworm infections appear to reduce IL-6 and TNF-α [45–48, 51–53], elevated levels of which have been associated with increased COVID-19 severity [64]. Whipworm infection, on the other hand, appears to be associated with increased TNF-α [49, 50], although this relationship seems to be age- and severity-dependent. Thus, research needs to address the following questions related to species-specific outcomes: Is there is variation is how STH species affect disease susceptibility? Which, if any, specific STH species are linked with the greatest reduction in COVID-19 virulence? And how does timing and intensity of specific infection types alter COVID-19 outcomes?

### How does timing of STH infection affect COVID-19 disease outcomes?

The mechanisms by which parasites regulate host immune function and immune responses appear to vary based on when individuals are exposed [8]. If STHs do mitigate COVID-19 severity, will it occur only in individuals who were infected with an STH during immune system development, or will adults who are infected later in life also benefit? Moreover, does infection intensity affect any resulting STH-linked immunoregulation? In other words, is a heavy STH infection required to dampen hyperinflammatory immune activity, or do light infections result in similar immune effects?

Likewise, are the anti-inflammatory effects of STH infection in response to COVID-19 only apparent for those currently infected with helminths, or are these same immunoregulatory effects evident in those with a history of STH infection (even if they are not currently infected)? How recently does an individual need to be exposed to STH infection to exhibit anti-inflammatory immunoregulation? To examine the immune effects of past STH exposure, future studies can examine COVID-19 symptom severity in groups of individuals (e.g. immigrant communities) who have transitioned at various ages from regions with widespread STH disease to areas with no endemic STH infections.

### Are there harmful interactions between STH and SARS-CoV-2 infections?

Evidence suggests that STH infections are immunosuppressive. Do these immune dampening effects of STH infections increase host susceptibility to SARS-CoV-2 infection? If so, are the anti-inflammatory effects discussed above enough to outweigh the negative outcomes that this immunosuppression might cause? Will STH infection reduce the efficacy of any eventual COVID-19 vaccine? Further, some have argued that the excessive immune activity linked with severe COVID-19 may also predispose individuals for infection with other pathogens, including parasites [111]. Do we therefore see a bidirectional relationship, with STH infections increasing SARS-CoV-2 infection risk and SARS-CoV-2 also increasing STH infection susceptibility? Longitudinal data collection is needed to parse out the directionality of interactions between SARS-CoV-2 and STH infections. Finally, some have argued that areas with limited medical infrastructure and endemic STH infection may suffer a setback in the control of STH infection as a result of disrupted intervention programs during the COVID-19 pandemic [112, 113]. It is therefore necessary to consider how shifts in resource allocation negatively affect STH prevention and treatment, possibly compounding any harmful interactions between the two infection types.

### Is there population-level variation in STH immune effects?

If any relationship is evident between STH and SARS-CoV-2 infection, how does this association vary between distinct populations? What lifestyle differences (e.g. national medical care system, typical diet, medication use, population density and demographics, smoking prevalence, etc.) may account for this variation? What underlying biological factors (e.g. blood type, intestinal microbiota diversity, previous exposure to other pathogens, etc.) may influence these interactions? How do interactions between STHs and the intestinal microbiota affect COVID-19 outcomes? And finally, how do the metabolic and nutritional costs associated with both STH infection and the microbiome affect nutritional status and disease prognosis?

## CONCLUSION

In conclusion, while little is definitively known given the novelty of the virus in humans, few studies have tested links between COVID-19 outcomes and co-existing parasitic infection. Very preliminary research suggests meaningful relationships between endemic STH infection and local SARS-CoV-2 risk/prognosis, but the effect of parasite infection on COVID-19 patterns likely varies by parasite species and based on host characteristics (e.g. other comorbidities, immune system competency, nutritional status, etc.) [66, 80, 83]. Because STHs typically infect vulnerable, marginalized and neglected populations, it is crucial that we work to understand the effects that these parasites may have on COVID-19 outcomes if we want to thoroughly understand the global impacts of SARS-CoV-2.

## Glossary

**Severe Acute Respiratory Syndrome Coronavirus 2 (SARS-CoV-2**). The novel coronavirus first identified in Wuhan, China in 2019 which has now spread globally, resulting in a prolonged pandemic

**Coronavirus Disease 2019 (COVID-19).** The disease caused by the virus SARS-CoV-2. Patients who develop severe COVID-19 exhibit a range of symptoms linked with hyperinflammatory immune responses, including acute respiratory distress that may result in death

**Angiotensin-converting enzyme 2 (ACE2).** A protein found on the surface of many different cell types and involved in cell function regulation. Coronaviruses, including SARS-CoV-2, have spike-like proteins that allow them to bind to ACE2, enter the cell, and disrupt normal ACE2 activity

**Cytokine**. A diverse group of small proteins involved in cell signaling and communication, making them essential in activating and coordinating immune responses. Specific types of cytokines include interleukins (IL), tumor necrosis factors (TNF), transforming growth factors (TGF), interferons (IFN), and C-Reactive Protein (CRP). Some cytokines increase immune activity and/or are proinflammatory (e.g. IL-1β, IL-5, IL-6, IL-9, IL-12, IL-17, IL-22, TNFα, CRP, IFN-γ), while others are immunosuppressive and/or anti-inflammatory (e.g. IL-4, IL-10, IL-13, IL-33, TGF-β), although the effects of cytokines can vary based on context

**Cytokine storm.** A severe inflammatory immune reaction initiated by the release of too many cytokines over a short period of time, leading to extensive host tissue damage

**Parasite.** An organism that derives some or all of its essential nutrients from a host, generally considered to be harmful to the host

**Macroparasite.** Parasites with life cycles that involve transmission stages, such as eggs and larvae, which take place outside of the host. In other words, macroparasites do not multiply within the host

**Mismatch paradigm.** A framework within Evolutionary Medicine that considers how mismatches between our ancestral environments (which shaped human evolved biology) and current lifestyles (e.g. altered sanitation, diet, and physical activity patterns) may contribute to poor health and disease risk

**Hygiene hypothesis.** The idea that high levels of sanitation lead to reduced pathogen exposure during key developmental periods, resulting in a lack of immune stimulation by evolutionarily relevant pathogens, subsequent immune dysregulation, and associated increases in allergies, asthma, and autoimmune conditions

**Old friends hypothesis.** Related to the Hygiene Hypotheses, this hypothesis focuses on pathogens and parasites that have long coevolutionary histories with humans, including microparasites and commensal bacteria. Infections with these pathogens may be particularly important for stimulating proper immune system development. Lack of infection with these ‘old friends’ is expected to lead to immune dysregulation and chronic inflammatory conditions

**Soil-transmitted helminths (STHs).** Intestinal parasitic worms that are spread through fecal-oral contamination and are estimated to infect more than a quarter of the global population. While often asymptomatic or mild, severe infection can result in nutritional deficiencies, mental and developmental delays, organ failure and death

**Type 2 (TH2) immunity.** Immune responses generally linked with parasitic infection, which promote immunotolerance through production of regulatory T and B cells, as well as anti-inflammatory cytokines. Without parasitic exposure, this type of immunity is associated with increased allergic phenotypes

**Regulatory T cells (Treg).** Lymphocytes (a type of white blood cell) that develop in the thymus and are pivotal in the suppression of other immune cells. They result in the production of anti-inflammatory cytokines and promote self-tolerance

**Regulatory B cells (Breg).** Lymphocytes (a type of white blood cell) that mature in the bone marrow and are pivotal in the suppression of other immune cells. Similar to Treg cells, they result in the production of anti-inflammatory cytokines and promote self-tolerance

**Type 1 (TH1) immunity.** The branch of the immune system commonly associated with viral and bacterial infection. These immune responses are linked with the production of pro-inflammatory cytokines and typically geared toward actively clearing infections. This branch of immunity is also implicated in autoimmune responses

**Type 17 (TH17) immunity.** A more recently discovered branch of the immune system associated with a highly inflammatory cytokine response. TH17 immune cells are involved in responses to extracellular pathogens, generally acting to recruit other immune cells to infected host tissue

**T helper cells (CD4+ T cells).** Mature T helper cells that differentiate into either TH1 or TH2 cells and steer immune cell production toward either branch of immunity. CD4+ T cells release cytokines that coordinate the activity of other immune cells

**Eosinophil.** A type of white blood cell that has proinflammatory and anti-viral effects. Higher levels of eosinophils are also associated with helminth infections, as they help eliminate the parasites during their larval stages. They are also elevated in COVID-19 patients
